# Seeding and Overseeding Native Hayseed Support Plant and Soil Arthropod Communities in Agriculture Areas

**DOI:** 10.3390/life10040038

**Published:** 2020-04-11

**Authors:** Elisa Cardarelli, Rodolfo Gentili, Francesca Della Rocca, Marta Zanella, Sarah Caronni, Giuseppe Bogliani, Sandra Citterio

**Affiliations:** 1 Department of Earth and Environmental Science, University of Pavia, Via Ferrata 9, 27100 Pavia, Italy; elisa.cardarelli@unipv.it (E.C.); fdellarocca@gmail.com (F.D.R.); marta.zanella00@gmail.com (M.Z.); giuseppe.bogliani@unipv.it (G.B.); 2 Department of Earth and Environmental Sciences, University of Milano-Bicocca, Piazza della Scienza 1, 20126 Milano, Italy; sarah.caronni@unimib.it (S.C.); sandra.citterio@unimib.it (S.C.)

**Keywords:** grassland, agriculture, functional diversity, QBS-ar, invasive alien species, species translocation, common ragweed, microarthropod community, native flora, ecosystem services

## Abstract

Using native seed mixtures to create or recover grassland habitats in rotation to crops or in strips surrounding fields is considered a cost-effective practice to enhance ecosystem resilience and agro-biodiversity. The aim of this research was to assess the effects of native hayseed mixtures on plant and microarthropod communities in an agricultural area of Northern Italy. Three different experimental treatments were set up. The first was a control (C) (i.e., non-seeded plots left to spontaneous vegetation succession after ploughing no deeper than 15 cm). The second, hayseed seeded (Hs) after ploughing no deeper than 15 cm. The third experimental treatment was hayseed overseeded (Ov) on the resident plant community after only a superficial harrowing. Ov plots exhibited the preeminent positive effects on the total productivity and quality of the grassland in terms of total vegetation cover, cover and richness of typical grassland species (i.e., *Molinio-Arrhenatheretea* species), and cover of legumes, grasses and perennial species. Moreover, Ov sites exhibited the highest abundance of microarthropod taxa and soil biological quality (QBS-ar) but only in spring, when the disturbance of ploughing negatively affected Hs and C plots. On the other hand, Hs sites showed a great reduction of invasive alien (i.e., *Ambrosia artemisiifolia* and *Artemisia verlotiorum*) and segetal weed species (i.e., *Capsella bursa-pastoris* and *Spergula arvensis*) in terms of cover. This study provides valuable indication on using hayseed mixtures to create grassland habitats as reservoir of native flora and soil biodiversity in agriculture areas.

## 1. Introduction

Over the last century, intensive agronomic practices have been one of the main drivers of the global biodiversity loss in agriculture areas [1], reducing the ability of agro-environments to provide ecosystems services [2]. The disturbance created by farming techniques (e.g., mowing, ploughing, massive use of fertilizers and pesticides) favors the colonization of weeds and invasive alien species able to displace native non-weed species [3,4,5]. On the other hand, this disturbance has negative impacts on above- and below-ground fauna [6,7,8,9]. As a consequence, a serious reduction of agroecosystem quality, in terms of biological community (plants, bacteria and arthropods) and of soil organic matter and carbon content has been registered [1].

In recent times, sustainable and multifunctional agricultural systems have managed to support high biodiversity levels (i.e., agro-biodiversity) which demonstrates a positive impact on ecosystem services and are useful to farmers and society [10]. It is well known that high levels of agro-biodiversity can increase the resilience of agroecosystems by reducing the risks coming from environmental changes and favoring mechanisms of adaptation to climate change such as a greater resistance to drought, pest outbreaks and pathogen transmission [11,12]. The sustainable management of agricultural systems implies the application of ecosystem-based practices supporting both biodiversity and crop production [10]. Such practices may include the following actions: (a) favoring landscape diversity by the maintenance of hedgerows, herb strips, riparian areas [2]; (b) reducing soil disturbance through no-till or minimum tillage practices [13] and through the seeding of a species-rich grass/legume intercrop which may increase diversity of the agriculture system [14]; (c) enhancing the presence and abundance of pest enemies in order to reduce the use of pesticides (pest management strategies) [15]; (d) maintaining and protecting species–species interactions in trophic networks [16]; (e) and maintaining high levels of wild pollinators [17].

Creating or recovering temporary and permanent grassland habitats in rotation to crops or surrounding fields by using native hayseed mixtures is considered a cost-effective environmental-friendly practice. It concomitantly enhances ecosystem resilience, increases soil quality and crop yield, favors pollinators, and contrasts segetal weeds and invasive alien species [7,18,19,20]. Indeed, herbaceous fields or strips may act as refugee for plants and wildlife from detrimental agronomic practices, by acting as a reservoir of native flora and soil biodiversity [21,22], and hosting natural enemies of crop pests [23].

Among native seed mixtures, hayseed is a species-rich mixture included in hay mowed from local meadows. Hayseed transfer is a well-known traditional agriculture practice [24]. In the past, farmers collected hay (including seed and plant residuals) from the floor of barns and sowed it on fields to establish new meadows and pastures, or to improve existing ones [25]. Recently, this management method has been successfully applied to control invasive species as a result of its mulching effect and to increase levels of competition [18,26].

In agricultural areas, an increase in species richness can be accomplished by using seeding or overseeding methods. While in the seeding method fields are subject to harrowing and ploughing, the overseeding foresees only harrowing [18]. Therefore, different levels of environmental conditions are assumed to be obtained from the two methods also in comparison with agriculture areas cultivated with main crops (e.g., cereals).

As far as we know, it is rare to find other studies where the different effects of seeding or overseeding methods for the creation of grassland in agriculture areas are simultaneously tested [27]. In addition, studies considering both plants and microarthropod communities are not common. Therefore, we hypothesized that seeding and overseeding methods for grassland establishment, in comparison to a control non-seeded area, may improve ecosystem components in agriculture areas.

The aim of this work was to assess the effects of seeding and overseeding hayseed mixtures on vegetation cover and structure, diversity levels (plants, ecological species group, and arthropods), and biological soil quality (QBS-ar) compared with no-seeded stands, in an agricultural area of Northern Italy (Padanian plain).

## 2. Materials and Methods

### 2.1. Study Area and Experimental Design

The study was performed in the Alto Milanese Park (359 ha), a protected area located in Northern Italy, approximately 30 km northwest of Milan (45°35′ N, 8°51′ E). The site is part of the local network of protected areas, instituted with the aim to preserve agriculture areas and peri-urban parks from urbanization, in order to improve the ecological connections among more strictly protected areas (i.e., natural parks and reserves) in the Lombardy region. In the park, cropped fields cover about 60% of the surface and are often surrounded by fallow fields (1.6%) and hedgerows (3.8%). Woodlands (17%) are dominated by *Prunus serotina* Ehrh. and *Robinia pseudoacacia* L. [28].

**Figure 1:**
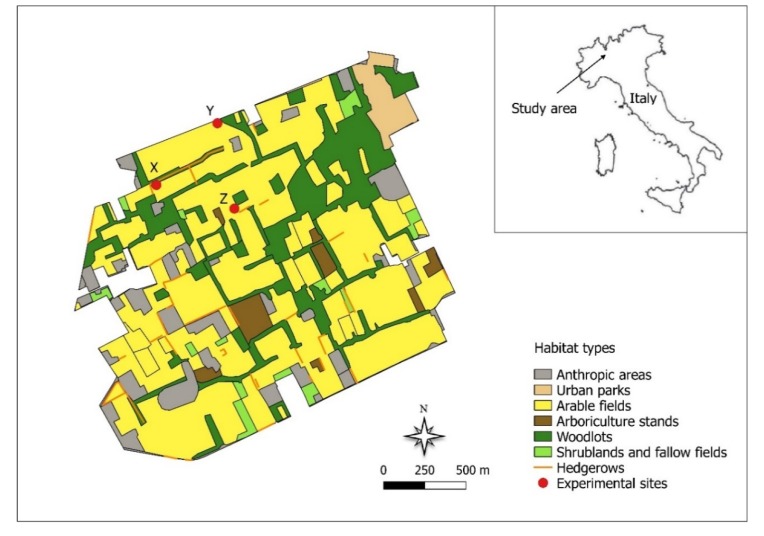
Location and land use of the study area in Northern Italy. Red dots indicate the experimental sites (X, Y, Z).

In 2014, three sites with comparable soil properties (Figure 1 and Table S1) were selected inside the park: (1) a short-rotation clover field (X); (2) an oat field (Y); and (3) a short-rotation meadow (Z). Each site contained three squared plots of 10 m × 10 m, separated by a 1 m buffer, for a total of nine plots. At each site, the following treatments were prepared in October 2014:

(a) control (C): plots left to spontaneous vegetation succession after harrowing and ploughing them no deeper than 15 cm;

(b) hayseed (Hs): plots seeded with hayseed at a density of about 20 g/m^2^ after harrowing and ploughing them no deeper than 15 cm. The hayseed used for this treatment was collected in June 2013 in a selected mesophilous grassland dominated by *Arrhenatherum elatius* (L.) Presl close to the study area (hereafter donor grassland). The most frequent species of the donor grassland and therefore of the mixture were: *A. elatius*, *Achillea millefolium* L., *Anthoxanthum odoratum* L., *Centaurea nigrescens* Willd., *Crepis capillaris* (L.) Wallr., *Holcus lanatus* L., *Poa pratensis* L., *Trifolium pratense* L. and *T. repens* L. Once dried, hayseed was prepared in accordance with the protocols of the Native Flora Centre of the Lombardy Region [25];

(c) overseeding hayseed (Ov): plots overseeded with hayseed at a density of about 20 g/m^2^ after only a superficial harrowing. The hayseed mixture used for this treatment was the same as the one used in Hs plots.

### 2.2. Data Collection

#### 2.2.1. Vegetation

In 2015, data on plant assemblages (species cover and composition) were collected in June, during the period of maximum flowering, in one 3 m × 3 m quadrat randomly chosen within each plot at least 1 m from the edge. Species cover was visually estimated by using a plastic quadrat of 1 m × 1 m subdivided in a grid 10 cm × 10 cm squared. The following parameters were recorded and organized in a database including information on.

(a) Vegetation cover (cumulative):
  - Total vegetation percent cover;
  - Percent cover of typical grassland species belonging to Molinio-Arrhenatheretea phytosociological class, based on the species composition of the donor grassland (Table S2);
  - Percent cover of segetal (ruderal) weed species (only infesting crop fields);
  - Percent cover of invasive alien species, listed according to the Lombardy region’s strategy for alien species [29]:
(b) Ecological groups:
  - Percent cover of the main plant families as a proxy of three main ecological groups of herb species following the classification used by Ma et al. [30] such as Poaceae (grasses), Asteraceae (forbs), Fabaceae (legumes), other families (other groups);
  - Life forms according to Raunkiaer [31].
(c) Species diversity:
  - α-diversity (species richness and Shannon diversity index [32]);
  - β-diversity (Whittaker diversity index [33]);
  - Richness of typical grassland species;
  - Richness of segetal weed species;
  - Richness of invasive alien species.

#### 2.2.2. Microarthropod Communities

Microarthropod data in terms of soil sampling, arthropod extraction and determination of biological forms was processed following the standard soil biological quality index methodology (QBS-ar) of Parisi et al. [34] (see also subsequent revisions [8,9]).

In 2015, soil samples were collected in spring (16th April and 6th May) and autumn (28th September and 24th October), during the peak activity of soil fauna [35]. In each plot, three random samples were collected with a cylindrical corer (10.5 cm diameter) inserted at a depth of 10 cm. Samples were placed in black fabric bags, labelled, and processed in the laboratory on the same day of collection to maximize organism survival and extraction. Soil fauna was extracted using Berlese-Tullgren funnels, with an extraction duration of five days. Microarthropods were mainly determined at the order level and, for two samples, one in spring (i.e., April) and one in autumn (i.e., September), the number of collected individuals was counted.

The community was characterized using individual abundance, calculated as the mean of the three samples collected in each plot, for the more representative taxa (i.e., with at least 30 individuals) for the April and September samples; richness within the plot, in terms of number of total taxa and number of euedaphic taxa (i.e., those well-adapted to the soil environment); and the QBS-ar index, for each month’s samples. QBS-ar is an index based on the biological form approach and assesses the adaptation of organisms to an edaphic habitat through an index (Eco-Morphological Index, EMI) that ranges from 1 to 20, with higher values assigned to microarthropod taxa that are better adapted to the soil environment. QBS-ar is the sum of the EMI scores of microarthropod groups recorded in the sample. It follows the concept that the higher the number of groups that are well-adapted to the edaphic habitat, the higher the soil quality.

### 2.3. Data Analysis

Differences in vegetation and microarthropod communities between treatments were tested by linear mixed effects models (LME). As for the models on vegetation cover and species diversity, treatment was fitted as a fixed factor, whereas for plant ecological groups, treatment and plant category (i.e., family or life form) were fitted as interacting fixed factors. As for models on QBS-ar and richness of microarthropod taxa and abundances, treatment and month were fitted as interacting fixed factors. Data on abundances of larvae and adult of Coleoptera and Diptera were tested separately, as different life stages have different adaptation to soil environment. In all models, the site (X, Y, Z) was fitted as a random effect in order to take into account the variability among sites due to their different characteristics (e.g., cropping history). When necessary, data were log-transformed to normalization.

All statistical analyses were performed using R version 3.6.1 [36] by means of the packages “nlme” [37] and, for post-hoc tests, “lsmeans” [38] and “multcomp” [39]. For more details, see Table S3.

## 3. Results

### 3.1. Vegetation

In total, 57 plant species were registered in the study area (no rare or protected species were detected). The dominant species in the treatments were *Ambrosia artemisiifolia* L. in C, *A. elatius* and *H. lanatus* in Hs, and *A. elatius*, *Artemisia verlotiorum* Lamotte, *T. pratense* and *T. repens* in Ov.

#### 3.1.1. Vegetation Cover

**Figure 2:**
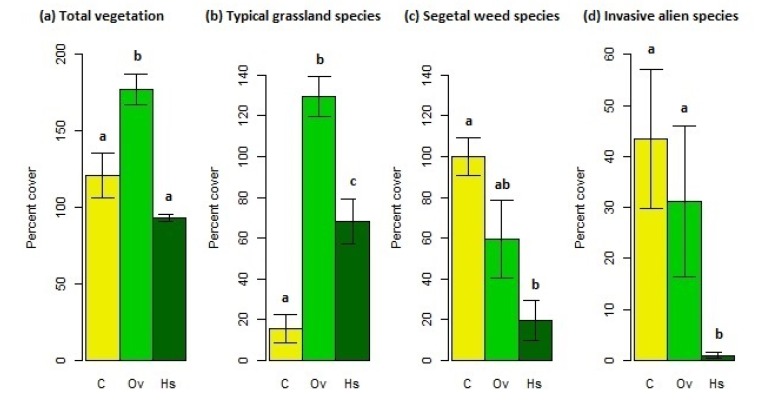
Cumulative percent cover (mean ± S.E.) of (**a**) total vegetation, (**b**) typical grassland species, (**c**) segetal weed and (**d**) invasive alien ones in control (C), overseeded (Ov) and hayseed (Hs) plots. Treatments with the same letters indicate no significant differences.

Total vegetation cover (cumulative) exhibited marked differences among the three treatments (F = 17.83, *p* < 0.01; Figure 2a; Table S4). As expected, Ov showed the highest total cover and was significantly different from C and Hs which exhibited the lowest value (no significant differences between C and Hs). The cover of the typical grassland (donor grassland composition) species varied across treatments (F = 119.96, *p* < 0.001; Figure 2b; Table S4): Ov once again showed the highest cover which was significantly different from Hs and C, in turn, Hs was significantly higher than C. Among the typical grassland species *A. elatius*, *H. lanatus* and *A. millefolium* were the most abundant or frequent. As for the cover of segetal weed and invasive alien species (Figure 2c,d; Table S4), the lowest values were observed in Hs in both cases (segetal weed species: F = 8.91, *p* = 0.034; invasive alien species: F = 6.78, *p* = 0.052). Among the weed species, *Capsella bursa-pastoris* (L.) Medik., *Papaver rhoeas* L. and *Spergula arvensis* L. were the most abundant or frequent, while among the invasive alien species *A. artemisiifolia* and *A. verlotiorum* were the most abundant.

#### 3.1.2. Ecological Groups of Species

**Figure 3:**
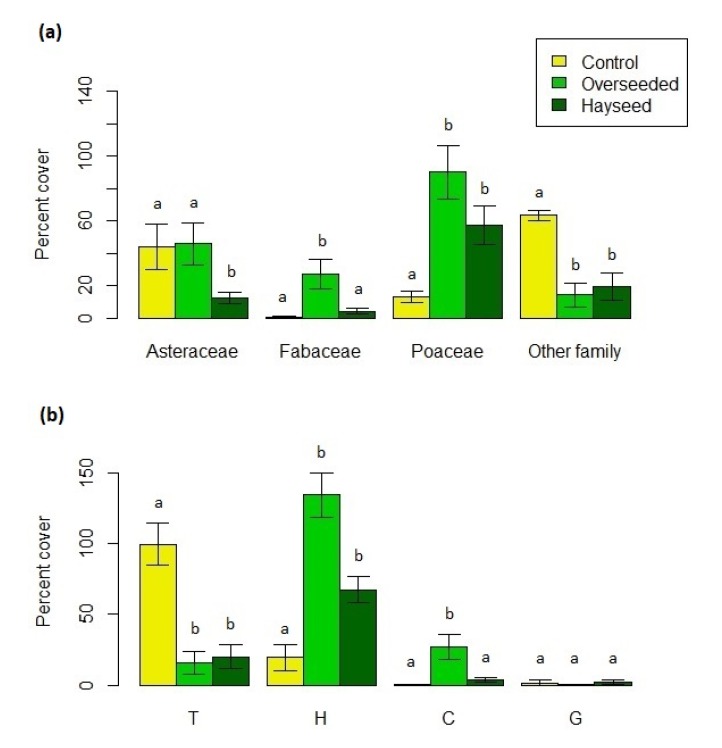
Cumulative percent cover (mean ± S.E.) of (**a**) the main families and of (**b**) life forms recorded in the three treatments. Abbreviation of life form: T = therophytes, H = hemicryptophytes, C = chamaephytes, G = geophytes. Treatments with the same letters indicate no significant differences (only differences among treatments within each family or life form are reported).

The main plant families greatly differed in the percent cover across treatments (F = 5.14, *p* = 0.015). In particular, the following significant differences were observed (Figure 3a; Table S4): Asteraceae showed the lowest amount in Hs; Fabaceae were recorded with the highest amount in Ov; Poaceae and other families showed, respectively, the lowest and highest amount in C.

Life forms greatly varied across treatments (F = 7.4, *p* < 0.01). In particular, the following significant differences were observed (Figure 3b; Table S4): therophytes cover was the highest in C compared with Ov and Hs; hemicryptophytes cover was higher in Ov and Hs than in C, while no differences between Ov and Hs were observed; chamaephytes cover was the highest in Ov compared to C and Hs, while no significant difference was detected between C and Hs; geophytes cover showed no significant differences among treatments.

#### 3.1.3. Plant Diversity

**Figure 4:**
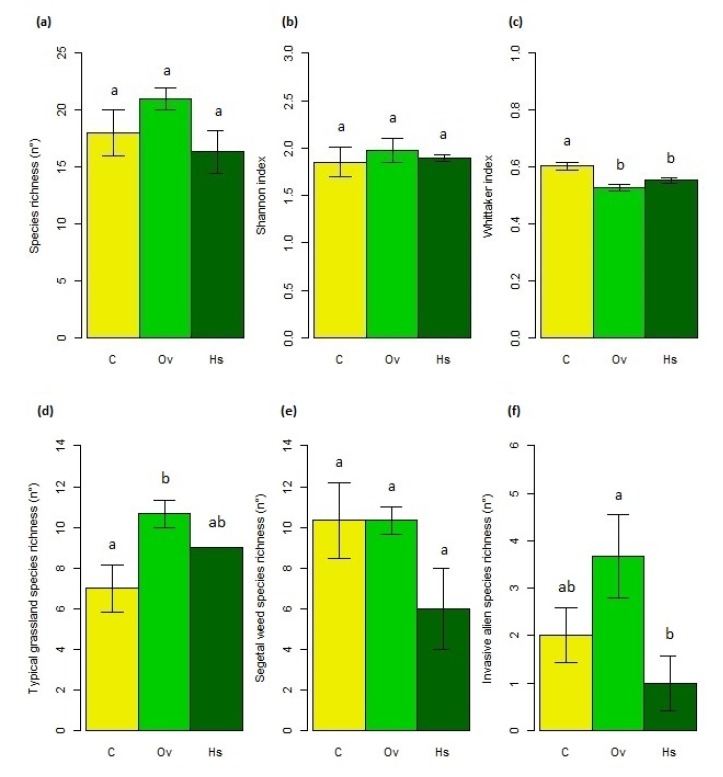
Mean (±S.E.) for (**a**) species richness, (**b**) Shannon index, (**c**) Whittaker index, and richness for (**d**) typical grassland species, (**e**) segetal weed and (**f**) invasive alien ones in control (C), overseeded (Ov) and hayseed (Hs) plots. Treatments with the same letters indicate no significant differences.

Overall diversity values did not exhibit significant differences among treatments (Figure 4a,b) even if, as a general trend, the highest absolute mean values of total richness and Shannon index were observed in Ov (mean species richness ± S.E.: C = 18 ± 2, Ov = 21 ± 1, Hs = 16.3 ± 1.86; mean Shannon index ± S.E.: C = 1.85 ± 0.16, Ov = 1.97 ± 0.13, Hs = 1.89 ± 0.03). However, significant differences were registered for the Whittaker index that were higher in C than Ov and Hs (F = 12.03, *p* = 0.02; Figure 4c; Table S4). Moreover, differences in richness of typical grassland species among treatment were recorded, even if marginally significant (F = 5.69, *p* = 0.068). In particular, a higher number of typical grassland species was observed in Ov compared with C (Figure 4d; Table S4). Finally, a higher number of invasive alien species was registered in Ov than in Hs (F = 7, *p* = 0.049; Figure 4f; Table S4).

### 3.2. Microarthropod Communities

In total, 12 taxa were collected in the study area from April to October, while 8288 individuals were counted in the samples collected in April (n = 3184) and September (n = 5104). Six taxa included euedaphic biological forms, even if most were represented by very few individuals (Symphyla = 22, Chilopoda = 10, Diplopoda = 2, Pauropoda = 1), with the exception of Acari (n = 5599) and euedaphic Collembola (n = 254). The dominant taxa were Acari and Collembola in all treatments and seasons, representing about 68% and 25% of the total community, respectively.

**Figure 5:**
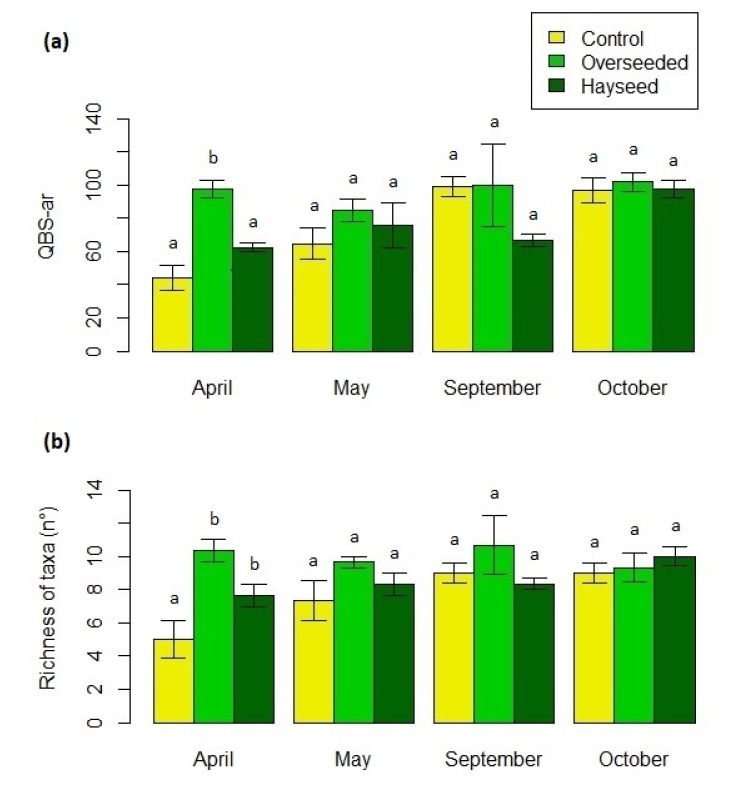
Mean (±S.E.) for (**a**) QBS-ar and (**b**) richness of taxa in the three treatments between April and October. Treatments with the same letters indicate no significant differences (only differences among treatments within each month are reported).

QBS-ar differed significantly among treatments during April (F = 11.62, *p* < 0.001) when Ov had higher values than C and Hs (Figure 5a; Table S4). No differences were detected forward in the season. Comparing treatment along the season (F = 10.76, *p* < 0.001), C showed a significant increase in QBS-ar values in September and October compared to April. As for richness, trends were similar to those of QBS. Ov and Hs hosted a significant higher number of taxa than C in April (F = 11.39, *p* < 0.001; Figure 5b; Table S4), while C showed a significant increase in richness during the season (F = 6.64, *p* < 0.01). No differences were recorded for euedaphic taxa richness among treatments and seasons, even if Diplopoda and Pauropoda were collected only in Ov but with negligible number.

**Figure 6:**
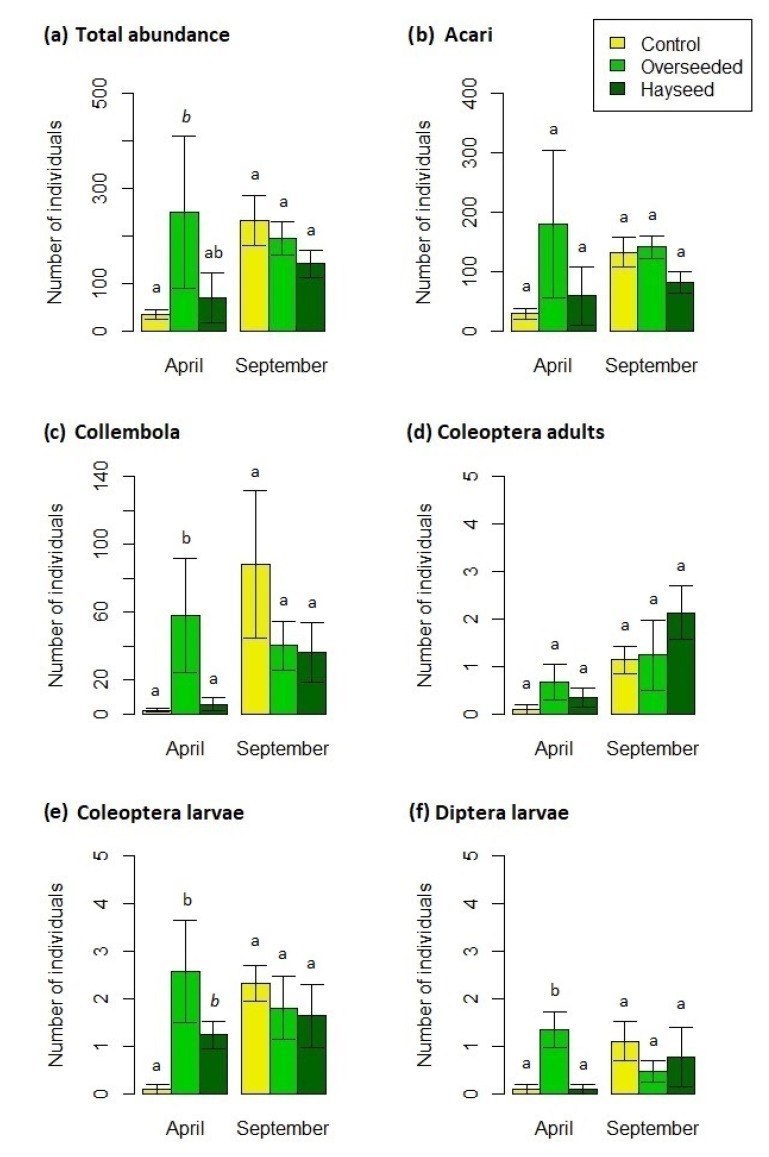
Number of individuals (mean ± S.E.) of (**a**) total microarthropods, (**b**) Acari, (**c**) Collembola, (**d**) Coleoptera adults, (**e**) Coleoptera larvae, and (**f**) Diptera larvae in the three treatments in April and September. Treatments with the same letters indicate no significant differences. Letters in italics indicate marginal significance (only differences among treatments within each month are reported).

The total abundance, even if marginally significant, was higher in Ov than C (F = 4.04, *p* = 0.052; Figure 6a; Table S4), and again C showed an increase from April to September (F = 9.22, *p* = 0.013); the same increasing trend from April to September was observed in C for Acari (F = 4.82, *p* = 0.053; Figure 6b; Table S4). Collembola number was higher in Ov than C and Hs in April (F = 11.22, *p* < 0.01; Figure 6c; Table S4) and increased significantly along the season in C and Hs (F = 27.56, *p* < 0.001). As for larvae, abundances decreased from Ov to Hs to C (Coleoptera: F = 8.27, *p* < 0.01; Diptera: F = 4.88, *p* = 0.03; Figure 6d,e; Table S4), while it increased along the season in C (Coleoptera: F = 16.22, *p* < 0.01; Diptera: F = 5.05, *p* = 0.049). Adult Coleoptera also increased along the season in C and Hs (F = 9, *p* = 0.013; Figure 6f; Table S4). No differences were recorded for Araneidae, Hemiptera and Thysanoptera.

## 4. Discussion

Our research shows that seeding and overseeding native plant mixtures in an agriculture area has positive effects on ecosystem components in terms of plant and microarthropod communities, compared with a control area left to spontaneous colonization of vegetation. Mainly, after about one-year observation, there was ecosystem improvements in seeding plant mixtures such as the amelioration of vegetation cover and structure, the increase of diversity levels (richness of typical grassland species and of arthropod taxa during the spring season, ecological species groups ratio) and of soil biological quality (microarthropod assemblages). However, seeding and overseeding had some different and complementary effects on the agroecosystem quality.

### 4.1. Vegetation

The three considered treatments greatly differed with regard to the dominant species. Control plots, subject to the ploughing disturbance and left to spontaneous vegetation succession, were dominated by the invasive alien species *A. artemisiifolia* which is very abundant in continental regions of Europe like Northern Italy, especially in open areas and crop fields [40]. Hayseed plots (Hs), as expected and desired, were dominated by *A. elatius* and *H. lanatus.* These are among the characteristic and frequent species of grasslands of the central and western Padanian plain [41]. Overseeding plots (Ov) instead, were dominated by both a mixture of typical grassland species (like hayseed treatment), by abundant covers of legumes (*Trifolium* spp., more abundantly than in Hs) and by the successional perennial invasive *A. verlotiorum*. In one way, *Trifolium* spp. are very common both for their high frequency in managed grasslands and for their common use in rotation crops. On the other hand, *A. verlotiorum* tends to colonize crop field margins rather than managed/abandoned fields because it generally prefers nutrient-rich, neutral and mesophilous soils with medium water availability. As underlined by Jermini and Schoenenberger [42], it also effectively invades agricultural fields and meadows newly created since it is favored by anthropogenic disturbance and land use change because of its ability to regenerate from rhizome fragments. This could be the possible explanation of its high cover in the Ov treatment.

Our results highlighted that overseeding, more so than seeding hayseed, improves vegetation cover and structure. Particularly, overseeding creates lower disturbance than seeding at the ground level. This favors the formation of a denser vegetation with increased abundance of typical grassland species with high frequency of legumes (Fabaceae) and perennial species such as chamaephytes and hemicryptophytes. Previous work highlighted how important it is to ensure the presence of a high percentage of legumes to maintain grassland quality since they have a positive effect on nitrogen pools by increasing above-ground biomass production [43]. In addition, different proportions of life forms (a good ratio between annual and perennial species) present in Ov can be linked to diversified species composition and ecological species’ groups (forbs, grass and legumes such as Asteraceae, Poaceae and Fabaceae). This can lead to different grassland productivity (i.e., above-ground biomasses; [44,45]) which, in our results, seemed to be the highest in Ov. In any case, Ov, at least in the first year after the grassland establishment, did not obstruct the colonization of an aggressive invasive alien species like *A. verlotiorum* which is a perennial late colonizer.

On the other hand, Hs reduced the incidence of invasive alien species colonization, both annuals and perennials, by filling empty spaces and reducing available niches [5]. A previous study performed in the same area ascertained the effectiveness of hayseed seeding or overseeding in controlling noxious alien species like *A. artemisiifolia*. Indeed, a lower number of plants and reduced inflorescences contribute to diminish the soil seed bank of this species and its pollen production [18,46].

The control plots, left to spontaneous colonization of vegetation after ploughing, exhibited the worst performance in terms of total vegetation cover and structure; cover of legumes, grasses and perennial (i.e., chamephytes and hemicryptophytes) species; as well as segetal weed abundance and cover.

Overall, our results are consistent with other studies about grasslands in agroecosystems which showed increased plant/ecological groups diversity. They also emphasized the key role of management in influencing many grassland good properties [47] such as vegetation cover, plant species composition, traits variability (ecological species’ group [2] and diversity [48,49]), forage quality and production [50].

### 4.2. Microarthropod Community

Soil in our study area hosted microarthropod communities dominated by taxa poorly or only partially adapted to edaphic habitats (i.e., epiedaphic and hemiedaphic), while well-adapted groups (i.e., euedaphic) were substantially represented by Acari and Collembola. Indeed, the microarthropod soil biological quality index, QBS-ar, showed values typical of agriculture ecosystems [9,13,51] (i.e., below or around the threshold of 93.7 as proposed by Menta et al. [12]) to separate high quality soil from poor ones. However, some differences existed between treatments, in particular during spring. Overseeded plots (Ov) had higher QBS-ar values, richness and abundances of microarthropods than control plots (C) and, in some cases (e.g., QBS-ar), this was also true for hay seeded plots (Hs). This is likely due to ploughing carried out to prepare soil in C and Hs plots in contrast to Ov ones, where only superficial harrowing took place. Tillage is well known to disrupt soil communities. In particular, QBS-ar is considered to show good sensitiveness to soil practices [8], often resulting in lower values in soils disturbed by tillage [13]. Moreover, Tabaglio et al. [6] reported a higher abundance of microarthropods in no-till soils than in conventional tillage, highlighting how mechanical operation strongly impacts suitability of habitat for soil fauna [52,53]. However, as observed in our study, the same authors reported that most of these differences disappear in autumn, suggesting that, given sufficient time without soil disturbance, arthropod numbers are able to recover within the growing season [21,54].

As for Hs soil in April, it is also interesting to note that, even if the quality was worse than Ov plots, it presented, in complex, communities with a higher richness of taxa than C plots. This is probably due to the vegetation cover which in early spring was more developed and structured in seeded plots (Ov and Hs) than in control ones [55]. The early seeding of a native species mixture of hayseed already demonstrated a faster establishment of grasses and herbs typical of grassland, also preventing the germination of invasive alien species [18]. Indeed, grass covering up the soil is another important factor in determining the presence of a rich soil community [8]. It is therefore logical conservative agriculture which applies the use of sustainable management including cover crops, promotes microarthropod assemblages [13,53].

In autumn, as previously stated, a recover of faunal assemblages in C and Hs plots was observed and differences between the treatments were no longer recorded, even if variations in cover of typical grassland, segetal weed and invasive alien species were registered. In particular, at the end of the growing season, segetal weeds/invasive aliens, above all the invasive *A. artemisiifolia*, dominated control plots in terms of percentage cover (see also [18]), while Ov and Hs plots registered the highest percent cover of grassland species. Many studies reported contrasting effects of invasive species on arthropods. Detritivores—which includes species from several taxa such as Collembola, Acari, Coleoptera and Diptera—benefit from plant invasion because of an increase in ground litter and decaying vegetation that can provide more feeding resources (leaves and litter) and favorable microclimatic conditions [56]. This effect on invertebrates was also detected by Qin et al. [57]. Specifically, for *A. artemisiifolia* they observed that the density of soil fauna was higher in plots dominated by the invasive alien species than in plots characterized by native flora. It is obvious that changes in plant composition not only had effects on soil fauna abundance but may have caused a shift in composition that could alter food web dynamics and plant-soil feedbacks, with consequences that deserve to be more deeply understood [56,58,59].

The authors are aware that the short time period of observation (one year) and the small sample size are limitations. However, the sampling took place on soil with comparable properties and the site was included in the statistical analyses, thus containing the site effect and supporting the reliability of the analyses. Further studies, involving more farms on a wider area, are desirable to disentangle long-term effects of the different treatments on plant and soil communities. These include seed germination intensity and seedlings survival, as well as intensity of plant growth in relation to the structure of microarthropod assemblages.

## 5. Management Implications

The use of hayseed seeding and/or overseeding is well-matched with the EU Directive 2078/92 regarding sustainable farming practices, which favor environmental amelioration and protection of natural resources. The creation of grasslands through the seeding or overseeding of native mixtures should be encouraged in agroecosystems as a sustainable management strategy and for enhancing their ecosystem functioning (e.g., aesthetic role, recreational function, maintenance of pollinators and soil fauna, purification of soil from pesticides and regularization of water regimes [21]). Moreover, the sowing of grasslands after a main crop (such as cereals) by using hayseed may avoid nitrogen and mineral leaching and stop soil erosion due to wind and precipitation during the winter season [60].

In this study, the importance of grassland as a reservoir of native flora (i.e., grassland species) and soil biodiversity (including ecological group diversity) is confirmed. Overseeding over the resident plant community results in better performances than hay seeding to create grassland habitats because it did not disrupt soil communities thanks to low-tillage. On the other hand, hay seeding inhibits the establishment of segetal weed and invasive alien species. In conclusion, the creation or recovery of grasslands using native plant mixtures is strongly recommended even if site-specific environmental characteristics are taken into account. Overseeding should be in general preferred, but in areas where segetal weed and invasive alien species are particularly widespread, hay seeding after ploughing could be the best solution.

## Supplementary Materials

The following are available online at https://www.mdpi.com/2075-1729/10/4/38/s1, Table S1: Soil characteristic in the three investigated sites, Table S2: Plant composition of donor grassland, Table S3: Linear Mixed Effects models (LME) specification, Table S4: Pairwise comparison by means of Tukey post-hoc test among treatments and months.
