# Supplementary material for: Seeding and Overseeding Native Hayseed Support Plant and Soil Arthropod Communities in Agriculture Areas
Source: Life (Basel). 2020 Apr 11;10(4):38. doi: 10.3390/life10040038 (PMC7235896; doi:10.3390/life10040038)
Supplement: Supplementary file 1 [file life-10-00038-s001.pdf]

life

MDPI

Supplementary Materials

Seeding or Overseeding native hay seed increase vegetation cover, functional diversity and soil quality in an agriculture area invaded by weeds

Cardarelli E. [1,#], Gentili R.[2,*,#], Della Rocca F. [1], Zanella M. [1], Caronni S. [2], Bogliani G. [1] and Citterio S. [2]

[1] Department of Earth and Environmental Science, University of Pavia, Via Ferrata 9, 27100 Pavia, Italy
[2] Department of Earth and Environmental Sciences, University of Milan-Bicocca, , Piazza della Scienza 1, 20126 Milano, Italy
* Correspondence: Gentili Rodolfo
# These authors contributed equally to this research

Table S1. Soil characteristic in the three investigated sites. Abbreviations = Texture: Sc: coarse sand, Sf: fine sand, Stot: total sand; Texture class: FS: sandy loam; Elements: C: carbon, Ntot: total nitrogen, C/N, S.O.M.: soil organic matter, CEC: cations exchange capacity, Cations: Ca^{++}, Mg^{++} K^{+}, BSR: base-cation saturation ratio, AvP: available phosphorus.

Analysis / Sites	Apparent texture					Texture class USDA	pH (H_2O)	pH (KCl)
	Sc(µm) 2000-100	Sf(µm) 100-50	Stot(µm) 2000-50	Silt (µm) 50-0,2	Clay (µm) <0,2			
	g/Kg							
X	551	97	648	279	73	FS	6,2	5,2
Y	495	100	595	321	84	FS	5,0	4,7
Z	522	120	642	263	95	FS	5,7	4,8

Analysis / Sites	Elements Elements Ca^{++} Mg^{++} K^{+}									
	C org.	S.O.M.	N tot.	C/N	CEC	Ca^{++}	Mg^{++}	K^{+}	BSR	AvP
	g/Kg				cmol(+)/Kg					mg/Kg
X	23	39	2	11,3	13,94	4,28	0,48	0,2	35,6	24
Y	30	51	2,95	10,1	11,38	1,45	0,33	0,03	15,9	51
Z	28	48	2,79	10,0	10,63	2,74	0,48	0,12	31,4	52

Table S2. Plant composition of the donor grassland.

Type of vegetation. *Arrhenatheretum elatioris* Br.-Bl. 1915

Site of collection: Olgiate Molgora (LC)

Diagnostic species:

Achillea millefolium subsp. *millefolium,*

Agropyron repens,

Anthoxanthum odoratum,

Bromus hordeaceus subsp. *hordeaceus,*

Cerastium glomeratum,

Centaurea nigrescens,

Dactylis glomerata,

Daucus carota,

Erigeron annuus,

Galium mollugo,

Medicago lupulina,

Pimpinella saxifraga,

Plantago lanceolata,

Poa pratensis,

Taraxacum officinale,

Trifolium sp pl.,

Veronica chamaedrys.

Rerference

Gusmeroli F., Della Marianna G., Parolo G. I prati della media Valtellina. Quaderni della Ricerca (Regione Lombardia), **2005**, 81, 5-25.

The complete list of the "Donor grasslands" (unedited data) is available at the following reference:

UNIMIB, CFA. Il contrasto ad Ambrosia con la semina di specie autoctone. Technical Report, Fondazione Cariplo, Bando Biodiversità, Milano, 2016.

CENTRO FLORA AUTOCTONA DELLA REGIONE LOMBARDIA - centroflora@parcobarro.it

Table S3. Linear Mixed Effects models (LME) specification. Fixed factors, log-trasformation (yes/no) and R packages used to analyze plant and microarthropod communities' differences among treatments (control, hayseeded and overseeded). The site (X, Y, Z) was always fitted as random effect. All LME models were performed with the package "nlme" [1], while post-hoc tests by means of the packages "lsmeans" [2] or "multcomp" [3]. Vegetation cover refers to cumulative percent, while abundance of microarthropod taxa to the mean of individuals collected in the three samples of each plot.

Variable (x)	log (x+1)	Fixed factors	Post-hoc test (package)
Vegetation			
total vegetation percent cover	no	treatment	multcomp
percent cover of typical grassland species	no	treatment	multcomp
percent cover of segetal weed species	no	treatment	multcomp
percent cover of invasive alien species	yes	treatment	multcomp
percent cover of main plant families	yes	treatment*family	lsmeans
percent cover of life form	yes	treatment*life_form	lsmeans
α-diversity	no	treatment	multcomp
β-diversity	no	treatment	multcomp
richness of typical grassland species	no	treatment	multcomp
richness of segetal weed species	no	treatment	multcomp
richness of invasive alien species	no	treatment	multcomp
Microarthropod communities			
QBS-ar	yes	treatment*month	lsmeans
richness of taxa	yes	treatment*month	lsmeans
richness of euedaphic taxa	yes	treatment*month	lsmeans
total abundance of microarthropods	yes	treatment*month	lsmeans
abundance of Acari	yes	treatment*month	lsmeans
abundance of Collembola	yes	treatment*month	lsmeans
abundance of Coleptera adults	yes	treatment*month	lsmeans
abundance of Coleoptera larvae	yes	treatment*month	lsmeans
abundance of Diptera larvae	yes	treatment*month	lsmeans
abundance of Araneidae	yes	treatment*month	lsmeans
abundance of Hemiptera	yes	treatment*month	lsmeans
abundance of Tysanoptera	yes	treatment*month	lsmeans

References:

[1] Pinheiro, J., Bates, D.; DebRoy, S.; Sarkar, D. R Core Team. Nlme: Linear and Nonlinear Mixed Effects Models. R package version 3.1-141, URL:https://CRAN.R-project.org/package=nlme, 2019.

[2] Lenth, R.V. Least-Squares Means: The R Package lsmeans. *J. Stat. Soft.*, **2016**, 69(1): 1–33.

[3] Hothorn, T.; Bretz, F.; Westfall, P. Simultaneous Inference in General Parametric Models. *Biom. J.*, **2008**, 50(3): 346–363.

Table S4. Pairwise comparison by means of Tukey post-hoc test (t or z test) among treatments and months, and significance of the differences. Only significant differences were reported. Abbreviations = Treatments: C = control, Ov = overseeded, Hs = hayseeded; Months: Apr = April, Sep = September, Oct = October.

Variable	pairwise comparison	t / z	p
Vegetation			
Total vegetation percent cover	Ov vs C	3.92	< 0.001
	Hs vs Ov	-5.86	< 0.001
Percent cover of typical grassland species	Hs vs Ov	-8.32	< 0.001
	Ov vs C	15.47	< 0.001
	Hs vs C	7.16	< 0.001
Percent cover of segetal weed species	Hs vs C	-4.22	< 0.001
Percent cover of invasive alien species	Hs vs C	-3.56	< 0.001
	Hs vs Ov	-2.60	0.025
Percent cover of main plant families			
Asteraceae	C vs Hs	2.75	0.03
	Ov vs Hs	2.81	0.027
Fabaceae	C vs Ov	-6.18	< 0.001
	Ov vs Hs	3.82	< 0.01
Poaceae	C vs Ov	-4.36	< 0.001
	C vs Hs	-3.30	< 0.01
other families	C vs Ov	3.74	< 0.01
	C vs Hs	2.98	0.018
Percent cover of life form			
therophytes	C vs Ov	3.62	< 0.01
	C vs Hs	2.93	0.02
hemicryptophytes	C vs Ov	-3.76	< 0.01
	C vs Hs	-2.58	0.043
chamephytes	C vs Ov	-4.86	< 0.001
	Ov vs Hs	3.05	0.016
Whittaker index	Ov vs C	-4.83	< 0.001
	Hs vs C	-3.15	< 0.01
Richness of typical grassland species	Hs vs C	3.37	< 0.01
Richness of invasive alien species	Ov vs Hs	-3.70	< 0.001

Table S5. Continued.

Variable	pairwise comparison	t / z	p
Microarthropod communities			
QBS-ar			
April	C vs Ov	-4.81	< 0.001
	Ov vs Hs	2.63	0.038
Control	Apr vs Sep	-4.89	< 0.001
	Apr vs Oct	-4.76	< 0.001
Richness of taxa			
April	C vs Ov	-4.74	< 0.001
	C vs Hs	-2.83	0.025
Control	Apr vs Sep	-3.86	< 0.01
	Apr vs Oct	-3.86	< 0.01
Total abundance of microarthropods			
April	C vs Ov	-2.60	0.063
Control	Apr vs Sep	-3.04	0.013
Abundance of Acari			
Control	Apr vs Sep	-2.22	0.053
Abundance of Collembola			
April	C vs Ov	-4.53	0.003
	Ov vs Hs	3.47	0.015
Control	Apr vs Sep	-5.25	< 0.001
Hayseeded	Apr vs Sep	-2.77	0.02
Abundance of Coleptera adults			
Control	Apr vs Sep	-3.00	0.013
Hayseeded	Apr vs Sep	-3.86	0.003
Abundance of Coleoptera larvae			
April	C vs Ov	-4.02	< 0.01
	C vs Hs	-2.55	0.069
Control	Apr vs Sep	-4.03	< 0.01
Abundance of Diptera larvae			
April	C vs Ov	-2.71	0.053
	Ov vs Hs	2.71	0.053
Control	Apr vs Sep	-2.25	0.049
